# A community change in the algal endosymbionts of a scleractinian coral following a natural bleaching event: field evidence of acclimatization

**DOI:** 10.1098/rspb.2008.0069

**Published:** 2008-03-18

**Authors:** A.M Jones, R Berkelmans, M.J.H van Oppen, J.C Mieog, W Sinclair

**Affiliations:** 1 Marine Molecular Genetics, Department of Molecular and Life Sciences, Central Queensland UniversityRockhampton, Qld 4702, Australia; 2 Australian Institute of Marine SciencePMB No. 3, Townsville MC, Qld 4810, Australia; 3 Department of Marine Benthic Ecology and Evolution, Biological Centre, University of GroningenKerklaan 30, 9750 AA Haren, The Netherlands

**Keywords:** *Symbiodinium*, bleaching, clade D, coral, thermal tolerance, acclimatization

## Abstract

The symbiosis between reef-building corals and their algal endosymbionts (zooxanthellae of the genus *Symbiodinium*) is highly sensitive to temperature stress, which makes coral reefs vulnerable to climate change. Thermal tolerance in corals is known to be substantially linked to the type of zooxanthellae they harbour and, when multiple types are present, the relative abundance of types can be experimentally manipulated to increase the thermal limits of individual corals. Although the potential exists for this to translate into substantial thermal acclimatization of coral communities, to date there is no evidence to show that this takes place under natural conditions. In this study, we show field evidence of a dramatic change in the symbiont community of *Acropora millepora*, a common and widespread Indo-Pacific hard coral species, after a natural bleaching event in early 2006 in the Keppel Islands (Great Barrier Reef). Before bleaching, 93.5% (*n*=460) of the randomly sampled and tagged colonies predominantly harboured the thermally sensitive *Symbiodinium* type C2, while the remainder harboured a tolerant *Symbiodinium* type belonging to clade D or mixtures of C2 and D. After bleaching, 71% of the surviving tagged colonies that were initially C2 predominant changed to D or C1 predominance. Colonies that were originally C2 predominant suffered high mortality (37%) compared with D-predominant colonies (8%). We estimate that just over 18% of the original *A. millepora* population survived unchanged leaving 29% of the population C2 and 71% D or C1 predominant six months after the bleaching event. This change in the symbiont community structure, while it persists, is likely to have substantially increased the thermal tolerance of this coral population. Understanding the processes that underpin the temporal changes in symbiont communities is key to assessing the acclimatization potential of reef corals.

## 1. Introduction

Coral reefs owe their success to the symbiosis between reef-building corals and intracellular, phototrophic dinoflagellates of the genus *Symbiodinium* (zooxanthellae) that supply up to 95% of the coral host's energy requirements (Muscatine 1990). Under stressful environmental conditions, such as abnormally high water temperatures in combination with high light, this symbiosis can break down and the algae are lost in a process known as ‘bleaching’. Such conditions have occurred on reefs globally (Hoegh-Guldberg 1999; Wilkinson 2004) and are predicted to become more frequent as a result of global warming (Donner *et al*. 2005; Hoegh-Guldberg *et al*. 2007). Therefore, coral bleaching is considered one of the biggest threats to coral reefs (Marshall & Schuttenberg 2006).

Nuclear ribosomal and chloroplast DNA markers show that the genus *Symbiodinium* is highly diverse. The genus is currently divided into eight distinct clades (categorized as A–H), each containing multiple subclades, strains or types (Coffroth & Santos 2005; Pochon *et al*. 2006; Stat *et al*. 2006). This level of genetic diversity appears to be matched by appreciable levels of physiological diversity within and between clades. For instance, symbiont types differ in their photosynthetic response to light (Iglesias-Prieto *et al*. 2004) and temperature stress (Robinson & Warner 2006). Reef-building corals can form associations with members of six of the eight *Symbiodinium* clades (A–D, F and G; reviewed by Baker 2003) and some of these associations seem to be more flexible than others (van Oppen *et al*. 2004). *Symbiodinium* C is the most common symbiont type in *Acropora* corals on the Great Barrier Reef (van Oppen *et al*. 2001; LaJeunesse *et al*. 2004; Smith 2004) and certain types within this clade have been shown to be particularly sensitive to heat stress (Berkelmans & van Oppen 2006). *Symbiodinium* clade D is common in *Acropora* corals on shallow and inshore reefs and has been shown to be relatively tolerant to high temperatures (Glynn *et al*. 2001; Baker 2004; Fabricius *et al*. 2004; van Oppen *et al*. 2005*b*; Ulstrup *et al*. 2006). Some corals are known to harbour multiple types within a single colony (Rowan & Knowlton 1995; Rowan *et al*. 1997; Ulstrup & van Oppen 2003), which may allow for changes in the relative abundances of each symbiont type under influence of the environment (symbiont ‘shuffling’). One way in which this change can occur is by the predominant, thermally sensitive symbiont population being replaced by a population of thermally tolerant symbionts that arise from the presence of less abundant ‘background’ symbionts (Baker 2003). As a result, the entire coral colony becomes more thermally tolerant. This acclimatization mechanism has been shown to occur in at least one population of *Acropora millepora* on the Great Barrier Reef after transplantation to a different thermal environment (Berkelmans & van Oppen 2006). However, only a few species of coral have been shown to shuffle their symbiont communities (Goulet & Coffroth 2003; Thornhill *et al*. 2003; Goulet 2006, 2007) and the longest symbiont monitoring study to date indicates stability rather than wholesale changes in symbiont communities (Thornhill *et al*. 2006). Symbiont ‘switching’, i.e. the acquisition of new symbionts from the surrounding environment (Baker 2003), may be another way by which corals can achieve a functional change in their predominant symbiont population, but so far this has not been demonstrated in scleractinian corals. Since bleaching is predicted to become more frequent as a consequence of climate change (Dunbar *et al*. 1994; Hoegh-Guldberg 1999; Hoegh-Guldberg *et al*. 2007), shuffling to more heat-resistant symbiont types may be an important acclimatization mechanism, but it must operate at the scale of populations and communities if reefs are to acclimatize and become more resistant to subsequent events (Buddemeier & Fautin 1993; Buddemeier *et al*. 2004). To date, this has not been shown to occur in a natural setting.

In this study, we characterize the *Symbiodinium* community in an inshore population of *A. millepora* and compare the *Symbiodinium* community in the same tagged colonies before and after a natural bleaching that took place in 2006. This is the first field study that follows changes in *Symbiodinium* genotypes in specific colonies over 3 years that includes a natural bleaching event. We show a dramatic shift in the symbiont community within this host population as a result of the disturbance, which is likely to have increased its thermal tolerance. We argue that if this shift is sustained and is community wide, the reefs in this area are likely to have substantially increased their capacity to withstand the next bleaching event.

## 2. Material and methods

### (a) Study site

Our study site is a reef flat adjacent to Miall Island (23°09′ S 150°54′ E), which is 1 of 15 islands in the Keppel Island group, in the southern inshore Great Barrier Reef. Miall Island, like many of the islands in this group, has an extensive reef flat on its leeward shore with an average coral cover of approximately 50%, dominated by colonies of the corymbose, Indo-Pacific stony coral *A. millepora* (van Woesik & Done 1997). The region suffered moderate to severe mass bleaching (more than 60% corals bleached) in February 2002 (Berkelmans *et al*. 2004) and severe bleaching in January/February 2006 (89% corals bleached; R. Berkelmans & A. M. Jones 2006, unpublished data).

### (b) Coral sampling

To determine the *Symbiodinium* community composition before the bleaching, 460 colonies were tagged on the reef flat at Miall Island between September 2004 and March 2005. A small (2–3 cm) branch was sampled from the central area of each colony and placed in a labelled bag for subsequent storage in 100% ethanol. Symbiont changes were monitored in a subset of 79 tagged colonies that survived the bleaching three and six months (May and August, respectively) after the bleaching event in January/February 2006. The subset of 79 colonies was chosen haphazardly from surviving colonies and comprised 58 with predominantly C2-type (no background types detected), 15 with predominantly D-type (no background types detected) and 6 with both C2 and D types present. To minimize confounding of temporal trends in symbiont community by intracolony variation in symbiont types, we sampled from the same area within each colony on each sampling occasion and ensured that only the tips of branches were used for DNA extraction. Mortality in the *A. millepora* population was assessed six months after the bleaching event in August 2006 by visually estimating the percentage of live and dead coral tissue on 159 haphazardly chosen tagged colonies using pre-bleaching photos of each colony as a reference.

### (c) Genotyping and sequencing

DNA was extracted from coral tissue based on the method of Wilson *et al*. (2002). A combination of single-stranded conformation polymorphism (SSCP) analysis, cloning and DNA sequencing was used for symbiont identification. The internal transcribed spacer 1 (ITS1) region was amplified as described by van Oppen *et al*. (2001). SSCP analysis was used to identify the predominant symbiont type in each colony and estimate the relative abundance of *Symbiodinium* types within each sample when more than one type was identified. Relative abundances of less than 5–10% are not detected using SSCP (Fabricius *et al*. 2004). SSCP bands that were faint compared with another more intense band in the same sample were identified as background and predominant types, respectively. The presence of two equally intense bands in the same sample was interpreted as the colony hosting equal amounts of each type. Fabricius *et al*. (2004) found that this was a reliable method for estimating the relative abundance of different *Symbiodinium* types. SSCP profiles were assigned to symbiont type by comparing to reference samples of known identity and by cloning and sequencing in the case of novel SSCP profiles. Phylogenetic analyses are described in detail in the electronic supplementary material.

### (d) Statistical analysis of symbiont community changes

Counts of colonies of *A. millepora* before the bleaching were analysed using a Pearson's chi-squared contingency table to compare the frequencies of colonies with different combinations of predominant symbiont types C2, C1 and clade D with the null hypothesis that there were no differences in the observed and expected cell frequencies.

In addition, two separate multinomial loglinear regressions were used to test for significant changes in the (i) predominant- and (ii) low-level background symbiont types in the subset of 79 colonies three and six months after bleaching with the null hypotheses that there were no differences in the log ratios of the observed and expected cell frequencies. Predominant symbiont types (C2, D and C1) and background types (C2, D, C1, D^*^, multiple types and no background types) before bleaching and three and six months after bleaching were fixed factors in the analyses, and cases were weighted by the number of colonies of each type. The parameter estimates derived from the multinomial loglinear regressions were used to show the nature of any significant changes. All statistical analyses were performed with SPSS v. 15.0.

## 3. Results

### (a) Symbiont diversity at Miall Island before and after bleaching

Before the bleaching in 2006, *A. millepora* at Miall reef associated predominantly with *Symbiodinium* type C2 (93.5%, *sensu* van Oppen *et al*. 2001) and to a much lesser extent with *Symbiodinium* clade D (3.5%) or mixtures of C2 and D (3.0%; *Χ*_1_^2^=398, *p*<0.001, *n*=460, figure 1*a*). Cloning and sequencing of five clade D and six clade C ITS1 PCR products (370 bp in length) showed that these differed by 1–6 bp within clades, which we assume represents intragenomic variation. By late February 2006 when bleaching was at its most intense, the relative difference in bleaching susceptibility between corals predominated by C2 and D was clearly evident, with the former bleaching white and the latter normally pigmented (figure 2). Tagged corals harbouring a mix of *Symbiodinium* C2 and D were mostly pale in appearance.

In May 2006, three months after the bleaching, a major shift to thermally tolerant type D and C1 symbiont communities occurred in the surviving colonies. Of 58 C2-predominant colonies monitored post-bleaching, 30 became predominant in type D symbionts (most without detectable levels of C2 symbionts; figure 1*b*). Type C1 was not detected in any of the SSCP gels of samples just before the bleaching. By May 2006, however, C1 became the predominant type in 11 out of the 58 colonies and was clearly evident as a background type in 11 out of 17 colonies that remained C2 predominant. C2-predominant colonies without other detectable background symbiont types (lower detection limit 5–10%) made up only 4 out of the 58 colonies three months after bleaching. Of the 15 original D-predominant colonies monitored post-bleaching, 12 retained their D predominance while 3 changed to C1 predominance. All six colonies that initially hosted C2 with background clade D became D predominant by May 2006. A variant of D which we called D^*^ was not apparent in any colonies prior to bleaching but was detectable at low levels in 10% of colonies after bleaching (figure 1*b*,*c*). The appearance of previously undetected C1 and D^*^ led to an increased diversity of symbiont types three months after bleaching.

By August 2006, six months after bleaching, the proportion of predominant symbiont types in each of the three initial groups of colonies (C2 or D predominant and C2 with D) remained stable, but there were substantial changes in the mix of background types. In the group that changed from C2 to D predominance, none had detectable background levels of C2 in May but C2 reappeared in five colonies in August. In addition, the other two groups also showed a slight increase in the background occurrence of C2 in August, possibly suggesting the start of a drift back to pre-bleaching C2 predominance. By contrast, more colonies had C1 in May compared with August while the abundance of D^*^ increased from May to August (figure 1*b*,*e*). The loglinear regressions showed that a significant change occurred in the predominant symbiont types of the colonies at Miall Island. The C2-predominant colonies were more likely to have changed to clade D predominance than to have remained unchanged or changed to C1 predominance in both May and August (*Z*=−15.0, *p*<0.001, d.f.=10). Type C2 colonies were more likely to occur with clade D than any other type or combination of types (C1, C2, D or D^*^) in May (*Z*=29.7, *p*<0.001, d.f.=70) and August (*Z*=34.4, *p*<0.001, d.f.=70).

While the symbiont community change in surviving colonies was dramatic (71% changed predominance from C2 to D or C1 (*n*=79)), selective mortality also played a substantial role in shifting the symbiont community in the coral population. Of 159 colonies monitored for survival, 147 were initially C2 predominant and of these, 54 colonies suffered 100% mortality and a further 34 suffered more than 50% partial mortality (figure 3). Only 1 of 15 colonies that were initially D predominant died. The difference in mortality between clades was statistically significant (*p*=0.043, *Χ*_1_^2^=4.1), confirming their differential thermal tolerance.

In terms of relative contribution to symbiont community change, selective mortality accounted for 37% of the change while altered symbiont-type predominance accounted for 42% of the change (*n*=159). Just over 20% of the original C2-predominant population survived and maintained C2 as their predominant symbiont (cf. 93.5% prior to bleaching).

## 4. Discussion

We have shown field evidence of a dramatic shift in the symbiont community in a reef-building coral as a result of bleaching. The balance of the symbiosis shifted from a predominant association between *A. millepora* and *Symbiodinium* type C2 to a predominance of type D and to a lesser extent to predominance of type C1. This shift resulted partly from a change of symbionts within coral colonies that survived the bleaching event (42%) and partly from selective mortality of the more bleaching-sensitive C2-predominant colonies (37%). While these numbers are event, population and location specific, they do confirm that several interrelated processes play a role in shaping reef symbiont communities after bleaching episodes (Baker 2003). We propose that symbiont shuffling is a more likely explanation for the observed shift in symbiont communities than switching (i.e. de novo uptake) because (i) all 14 colonies that harboured low levels of D-type symbionts prior to the bleaching event survived and changed from C2 to D predominance, (ii) SSCP analysis is known to lack the sensitivity to detect symbiont types at a relative abundance of less than 5–10% and (iii) cloning and sequencing a subset of samples before bleaching revealed D and C1 below the detection limits of SSCP, the presence of which predicted their appearance after bleaching if shuffling was the mechanism of change. This is supported by the observation of novel symbiont types three and six months after the bleaching. Although de novo uptake cannot be ruled out, mathematical modelling of the recovery of symbiont populations after bleaching suggests that such rapid changes are more easily explained by upward and downward regulations of existing symbiont populations (Jones & Yellowlees 1997).

As a direct result of the shift in symbiont community, the Miall Island *A. millepora* population is likely to have become more thermo-tolerant. We base this conclusion on the experimental evidence of Berkelmans & van Oppen (2006) who found that differences in thermal tolerance in *A. millepora* from the same area is driven by symbiont type rather than the host coral. Furthermore, a shift from bleaching-sensitive type C2 to clade D increased the thermal tolerance of this species by 1–1.5°C. These findings are supported by our observation of differential bleaching susceptibility between C2- and D-predominant colonies during the 2006 bleaching event (figure 2). We suggest that *A. millepora* colonies that host predominantly C1-type symbionts are also more thermally tolerant than their counterparts with C2. Unbleached colonies of the staghorn coral *A. formosa* sampled in February 2006 at Miall Island harboured predominantly C1 symbionts whereas white-bleached colonies of this species hosted C2. These observations, together with the high occurrence of C1 in acroporid corals (van Oppen *et al*. 2001) at one of the most thermo-tolerant reefs on the Great Barrier Reef (Berkelmans 2002), suggest that C1 may confer thermal tolerance to some species, just like D-type symbionts. Given the direct experimental evidence of increased thermal tolerance of *A. millepora* with D-type symbionts and the circumstantial evidence of similar thermal tolerance in this species with C1-type symbionts, the symbiont community change documented in this study is therefore likely to have resulted in increased thermal resistance for the majority of the *A. millepora* population. If the symbiont community drifts back to C2 predominance, the increased thermal tolerance will be lost. A drift back to pre-bleaching symbiont types was suggested for *Montastraea annularis* in the Florida Keys (Thornhill *et al*. 2006), and there are signs of a similar drift back to pre-bleaching C2 predominance in this study six months after bleaching.

Our results strongly support the reinterpreted adaptive bleaching hypothesis of Buddemeier *et al*. (2004), which postulates that a continuum of changing environmental states stimulates the loss of bleaching-sensitive symbionts in favour of symbionts that make the new holobiont more thermally tolerant. However, such a change may come at a physiological cost such as loss of photosynthetic efficiency (Rowan 2004) leading to lower energy reserves (Hoogenboom *et al*. 2006; Loram *et al*. 2007) and slower growth (Little *et al*. 2004). Our field observations provide the first extensive colony-specific documentation and quantification of temporal symbiont community change in the field in response to temperature stress, suggesting a population-wide acclimatization to increased water temperatures. If this shift is sustained and extends to other species, the reefs in this area are likely to have substantially increased their capacity to withstand the next bleaching event. However, at this stage, it is unknown whether the increased thermal tolerance, even if it persists, will necessarily translate into increased reef resilience, particularly if growth and carbonate accretion are depressed to levels whereby bioerosion outweighs net accretion.

This study highlights the importance of improving our understanding of multi-clade symbiotic partnerships (Baker & Romanski 2007). Our results show an increase in the diversity of symbionts after bleaching together with a considerable change in the make-up of the symbiont community within individual colonies over time scales as short as three months. This increase in the diversity and variation of symbionts has not been previously shown following a bleaching event. Most studies that have followed the *Symbiodinium* community during bleaching (Glynn *et al*. 2001; Guzman & Cortes 2001; Baker 2003; Van Woesik *et al*. 2004) have not used molecular techniques sensitive enough to detect the low-density symbiont genotypes and genetic variations of rDNA types (Apprill & Gates 2007). A recent study has shown that the majority of scleractinian corals are likely to harbour symbiont types at levels that are undetectable using electrophoretic genetic techniques (Mieog *et al*. 2007), suggesting that symbiont flexibility may also be more common than previously thought. Subtle seasonal and spatial shifts in symbiont populations that occur as a result of even minor changes in environmental variables such as temperature and light may underwrite the more permanent, climate-driven shifts following dramatic bleaching events (Thornhill *et al*. 2006). Smith (2005) found that four months before a major bleaching event in early 2002, 20 out of 20 *A. millepora* colonies at Miall Island were predominant in type C2, while van Oppen *et al*. (2005*a*) found that five months after the 2002 bleaching event, 6 out of 19 were predominant in type D. Although the sample sizes in these studies are small, these results suggest that the *A. millepora* symbiont community underwent a similar shift towards clade D predominance as a result of the 2002 bleaching event and then drifted back to C2 predominance 4 years later just prior to the 2006 bleaching event. This poses the question of why some coral population retain thermally tolerant symbionts while others revert back to former sensitive types. Baird *et al*. (2007) hypothesize that symbiont community shuffling to clade D may persist only as a result of enduring changes in environmental conditions, e.g. repeated warm summers. This may be evident at Magnetic Island, where temperatures exceed 30.5°C during most summers (Berkelmans 2002) and *A. millepora* have harboured exclusively clade D symbionts over many years (van Oppen *et al*. 2001; Berkelmans & van Oppen 2006). Conditions similar to those currently occurring at warm reefs such as Magnetic Island have been projected to occur on in the southern Great Barrier Reef by 2020–2030 (Done *et al*. 2003). Understanding the role of these backgrounds symbionts and the process and conditions under which they are up- and downregulated is the key to assessing the acclimatization potential of coral reefs and their ability to withstand future thermal stress events in an era of climate change.

## Figures and Tables

**Figure 1:**
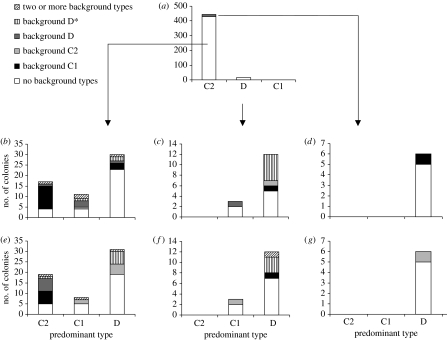
*Acropora millepora* colonies sampled (*a*) prior to bleaching between September 2004 and March 2005 (*n*=460), (*b*–*d*) three months after bleaching in May 2006 (*n*=79) and (*e*–*g*) six months after bleaching in August 2006 (*n*=79) at Miall Island reef (Keppel Islands, southern Great Barrier Reef). (*b*,*e*) Original C2 type, *n*=58, (*c*,*f*) original D type, *n*=16, (*d*,*g*) original C2/D type, *n*=6.

**Figure 2:**
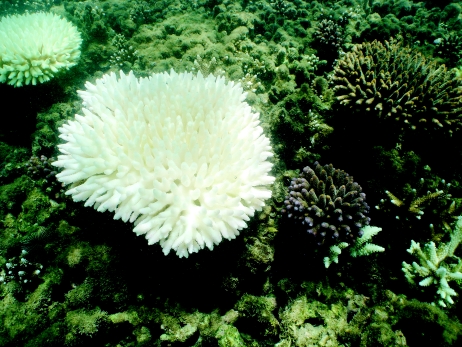
A stark landscape of 100% bleached *A. millepora* colonies predominated by *Symbiodinium* clade C2 and unbleached colonies with clade D symbionts at Miall Island reef in the Keppel region of the southern Great Barrier Reef during the January/February 2006 summer bleaching confirm the differential bleaching susceptibility of corals with these symbiont types.

**Figure 3:**
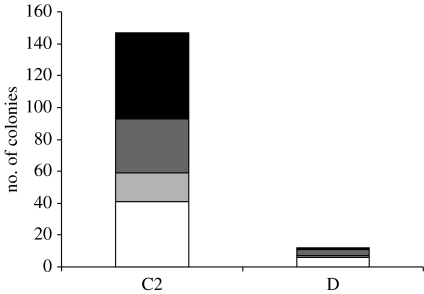
Summary of partial mortality in tagged *A. millepora* colonies at Miall Island (*n*=159). 37% of colonies with type C2 suffered 100% mortality compared with only 8% of clade D colonies (*Χ*_1_^2^=4.1, *p*=0.043). D, colonies with predominantly clade D; C2, colonies with predominantly type C2; and C2/D, colonies with type C2 and clade D. Within-colony mortality: black, 100%; dark grey, 51–99%; light grey, 11–50%; white, less than 10%.

## Supplementary Material

Additional Material The following is a supplementary material related to the paper by Jones *et al.* 2008 A community change in the algal endosymbionts of a scleractinian coral following a natural bleaching event: field evidence of acclimatization Proc. R. Soc. Lond. B. The material describes the methods used to distinguish the Symbiodinium types found at Miall Island during a study of symbiont community change following bleaching and describes their phylogenetic relationships. The material compares the symbiont types from this study with several types for which DNA sequences are available in Genbank. For this purpose the ITS1, the ITS2 and the complete ITS region is used

## References

[bib1] Apprill A.M, Gates R.D (2007). Recognizing diversity in coral symbiotic dinoflagellate communities. Mol. Ecol.

[bib2] Baird A.H, Cumbo V.R, Leggat W, Rodriguez-Lanetty M (2007). Fidelity and flexibility in coral symbioses. Mar. Ecol. Prog. Ser.

[bib3] Baker A.C (2003). Flexibility and specificity in coral-algal symbiosis: diversity, ecology, and biogeography of *Symbiodinium*. Annu. Rev. Ecol. Evol. Syst.

[bib4] Baker A.C, Rosenberg E, Loya Y (2004). Symbiont diversity on coral reefs and its relationship to bleaching resistance and resilience. Coral health and disease.

[bib5] Baker A.C, Romanski A.M (2007). Multiple symbiotic partnerships are common in scleractinian corals, but not in octocorals: comment on Goulet (2006). Mar. Ecol. Prog. Ser.

[bib6] Berkelmans R (2002). Time-integrated thermal bleaching thresholds of reefs and their variation on the Great Barrier Reef. Mar. Ecol. Prog. Ser.

[bib7] Berkelmans R, van Oppen M.J.H (2006). The role of zooxanthellae in the thermal tolerance of corals: a ‘nugget of hope’ for coral reefs in an era of climate change. Proc. R. Soc. B.

[bib8] Berkelmans R, De'ath G, Kinninmonth S, Skirving W.J (2004). A comparison of the 1998 and 2002 coral bleaching events on the Great Barrier Reef: spatial correlation, patterns, and predictions. Coral Reefs.

[bib9] Buddemeier R.W, Fautin D.G (1993). Coral bleaching as an adaptive mechanism: a testable hypothesis. Bioscience.

[bib10] Buddemeier R.W, Baker A.C, Fautin D.G, Jacobs J.R, Rosenberg E, Loya Y (2004). The adaptive hypothesis of bleaching. Coral health and disease.

[bib11] Coffroth M.A, Santos S.R (2005). Genetic diversity of symbiotic dinoflagellates in the genus *Symbiodinium*. Protist.

[bib12] Done, T., Whetton, P., Jones, R. J., Berkelmans, R., Lough, J. M., Skirving, W. & Woolridge, S. 2003 Global climate and coral bleaching on the Great Barrier Reef. Final report to the State of Queensland Greenhouse Task Force, 33.

[bib13] Donner S.D, Skirving W, Little C.M, Oppenheimer M, Hoegh-Guldberg O (2005). Global assessment of coral bleaching and required rates of adaptation under climate change. Global Change Biol.

[bib14] Dunbar D.B, Wellington G.M, Colgan M.W, Glynn P.W (1994). Eastern Pacific sea surface temperatures since 1600 A.D.: the d^18^O record of climate variability in Galapagos corals. Paleooceanography.

[bib15] Fabricius K.E, Mieog J.C, Colin P.L, Idip D, van Oppen M.J (2004). Identity and diversity of coral endosymbionts (zooxanthellae) from three Palauan reefs with contrasting bleaching, temperature and shading histories. Mol. Ecol.

[bib16] Glynn P.W, Mate J.L, Baker A.C, Calderon M.O (2001). Coral bleaching and mortality in Panama and Ecúador during the 1997–1998 El Niño-Southern Oscillation event: spatial/temporal patterns and comparisons with the 1982–1983 event. Bull. Mar. Sci.

[bib17] Goulet D (2006). Most corals may not change their symbionts. Mar. Ecol. Prog. Ser.

[bib18] Goulet D (2007). Most scleractinian corals and octocorals host a single symbiotic zooxanthella clade. Mar. Ecol. Prog. Ser.

[bib19] Goulet D, Coffroth M.A (2003). Genetic composition of zooxanthellae between and within colonies of the octocoral *Plexaura kuna* based on small subunit rDNA and multilocus DNA fingerprinting. Mar. Biol.

[bib20] Guzman H.M, Cortes J (2001). Changes in reef community structure after fifteen years of natural disturbances in the eastern Pacific (Costa Rica). Bull. Mar. Sci.

[bib21] Hoegh-Guldberg O (1999). Climate change, coral bleaching and the future of the world's coral reefs. Mar. Freshw. Res.

[bib22] Hoegh-Guldberg O (2007). Coral reefs under rapid climate change and ocean acidification. Science.

[bib23] Hoogenboom M.O, Anthony K, Connolly S.R (2006). Energetic cost of photoinhibition in corals. Mar. Ecol. Prog. Ser.

[bib24] Iglesias-Prieto R, Beltrán V.H, LaJeunesse T.C, Reyes-Bonilla H, Thomé P.E (2004). Different algal symbionts explain the vertical distribution of dominant reef corals in the eastern Pacific. Proc. R. Soc. B.

[bib25] Jones R.J, Yellowlees D (1997). Regulation and control of intracellular algae (zooxanthellae) in hard corals. Proc. R. Soc. B.

[bib26] LaJeunesse T.C, Bhagooli R, Hidaka M, deVantier L, Done T, Schmidt G.W, Fitt M.K, Hoegh-Guldberg O (2004). Closely related *Symbiodinium* spp. differ in relative dominance in coral reef host communities across environmental, latitudinal and biogeographic gradients. Mar. Ecol. Prog. Ser.

[bib27] Little A.F, van Oppen M.J.H, Willis B.L (2004). Flexibility in algal endosymbioses shapes growth in reef corals. Science.

[bib28] Loram J.E, Trapido-Rosenthal H.G, Douglas A.E (2007). Functional significance of genetically different symbiotic algae *Symbiodinium* in a coral reef symbiosis. Mol. Ecol.

[bib29] Marshall P, Schuttenberg H.Z, Phinney J.T, Hoegh-Guldberg O, Kleypas J, Skirving W, Strong A.E (2006). Adapting coral reef management in the face of climate change. Coral reefs and climate change: science and management.

[bib30] Mieog J.C, Van Oppen M.J.H, Cantin N, Stam D.W.T, Olsen J.L (2007). Real-time PCR reveals a high incidence of *Symbiodinium* clade D at low levels in four scleractinian corals across the Great Barrier Reef: implications for symbiont shuffling. Coral Reefs.

[bib31] Muscatine L, Dubinsky Z (1990). The role of symbiotic algae in carbon and energy flux in reef corals. Coral reefs.

[bib32] Pochon X, Montoya-Burgos J.I, Stadelmann B, Pawlowski J (2006). Molecular phylogeny, evolutionary rates, and divergence timing of the symbiotic dinoflagellate genus *Symbiodinium*. Mol. Phylogenet. Evol.

[bib33] Robinson J.D, Warner M.E (2006). Differential impacts of photoacclimation and thermal stress on the photobiology of for different phylotypes of *Symbiodinium* (*Pyrrhophyta*). J. Phycol.

[bib34] Rowan R (2004). Thermal adaptation in reef coral symbionts. Nature.

[bib35] Rowan R, Knowlton N (1995). Intraspecific diversity and ecological zonation in coral-algal symbiosis. Proc. Natl Acad. Sci. USA.

[bib36] Rowan R, Knowlton N, Baker A.C, Jara J (1997). Landscape ecology of algal symbionts creates variation in episodes of coral bleaching. Nature.

[bib37] Smith, C. 2004 The molecular and genetic basis for variation in thermal tolerance in a common reef-building species on the inshore Great Barrier Reef. PhD thesis.

[bib37a] Smith, C. 2005 The role of genetic and environmental variation on thermal tolerance of a reef-building coral, *Acropora millepora* PhD Thesis, University of Queensland.

[bib38] Stat M, Carter D, Hoeegh-Guldberg O (2006). The evolutionary history of *Symbiodinium* and scleractinian hosts—symbiosis, diversity, and the effect of climate change. Perspect. Plant Ecol. Evol. Syst.

[bib39] Thornhill, D. J., LaJeunesse, T. C., Schmidt, G. W. & Fitt, W. K. 2003 Change and stability of symbiotic dinoflagellates in reef building corals. In *Seventh Int. Conf. on Coelenterate Biology, Lawrence*

[bib40] Thornhill D, LaJeunesse T, Kemp D, Fitt W, Schmidt G (2006). Multi-year, seasonal genotypic surveys of coral-algal symbioses reveal prevalent stability or post-bleaching reversion. Mar. Biol.

[bib41] Ulstrup K.E, van Oppen M.J.H (2003). Geographic and habitat partitioning of genetically distinct zooxanthellae (*Symbiodinium*) in *Acropora* corals on the Great Barrier Reef. Mol. Ecol.

[bib42] Ulstrup K, Ralph P, Larkum A, Kühl M (2006). Intra-colonial variability in light acclimation of zooxanthellae in coral tissues of *Pocillopora damicornis*. Mar. Biol.

[bib43] van Oppen M.J.H, Palstra F.P, Piquet A.M.T, Miller D.J (2001). Patterns of coral–dinoflagellate associations in *Acropora*: significance of local availability and physiology of *Symbiodinium* strains and host–symbiont selectivity. Proc. R. Soc. B.

[bib44] van Oppen M.J.H, Little A, Willis B.L (2004). Algal shuffling helps corals cope with environmental change. Australas. Sci.

[bib45] van Oppen M.J.H, Mahiny A, Done T (2005a). Geographic patterns of zooxanthella types in three coral species on the Great Barrier Reef sampled after the 2002 bleaching event. Coral Reefs.

[bib46] van Oppen M.J.H, Mieog J.C, Sanchez C.A, Fabricius K.E (2005b). Diversity of algal endosymbionts (zooxanthellae) in octocorals: the roles of geography and host relationships. Mol. Ecol.

[bib47] van Woesik R, Done T.J (1997). Coral communities and reef growth in the southern Great Barrier Reef. Coral Reefs.

[bib48] Van Woesik R, Irikawa A, Loya Y, Rosenberg E, Loya Y (2004). Coral bleaching: signs of change in southern Japan. Coral health and disease.

[bib49] Wilkinson C.R (2004). Status of Coral Reefs of the World: 2004.

[bib50] Wilson K (2002). Genetic mapping of the black tiger shrimp *Penaeus monodon* with amplified fragment length polymorphism. Aquaculture.

