# Supplementary material for: A community change in the algal endosymbionts of a scleractinian coral following a natural bleaching event: field evidence of acclimatization
Source: Proc Biol Sci. 2008 Mar 18;275(1641):1359–65. doi: 10.1098/rspb.2008.0069 (PMC2367621; doi:10.1098/rspb.2008.0069)
Supplement: Supplementary file 1 [file rspb20080069s01.doc]

Supplementary material

Phylogenetic analysis of *Symbiodinium* ITS1

Following amplification of the ITS1 region and SSCP analysis, four different bands were observed (Fig. 1), and the ITS1 region of 8 representative samples were cloned and/or sequenced. Amplification products of about 370 bp length were obtained for all samples. Sequences were compared and aligned to closely matching sequences in Genbank using BioEdit (version 7.0) and CLUSTER W (version 1.7, Thomps*on et a*l. 1994). Primer sequences were removed from the alignments before analysis. Phylogenetic analyses were conducted using the Maximum Parsimony (MP) method (Saitou & Nei 1987) in PAUP (Version 4.0b10 for 32-bit Microsoft Windows). Either exhaustive or branch-and-bound searches were conducted, with gaps treated as a fifth base. Support for MP clusters was tested by bootstrap analysis with 1000 replicates (heuristic search). Type C and type D ITS1 sequences were analysed separately because this region is virtually un-alignable among clades.

The *Symbiodinium* ITS1 clade C alignment consisted of 253 alignment positions and 13 taxa (Taxa for which the ITS1 sequences were obtained for this study are shown in Table 1). Six positions were variable, two of which were parsimony informative. The *Symbiodinium* ITS1 clade D alignment consisted of 8 taxa and 329 characters, six of which were variable but not parsimony-informative. The clade D sequences obtained from the Keppel island *A. millepora* colonies are identical or very similar to clade D obtained from *A. millepora* at other Great Barrier Reef locations (van Opp*en et a*l. 2001, accession number EU024793). Phylogenetic analysis distinguished the two subclades C1 and C2 within clade C (van Opp*en et a*l. 2001, Berkelmans & van Oppen 2006) as well as a few other sequence variants (Fig. 2 a). There was one clade D variant (named type D*) that appeared on SSCP in a number of samples before the bleaching (Fig. 1). Sequence analysis of the clone matching the D* SSCP profile revealed a single base pair change from the most common type D variant (Fig. 2 b).

Cloning of samples that appeared to harbour one symbiont type (according to SSCP) in some samples revealed low-abundance background types that had missed detection (Fig. 3). For instance, nineteen clones of sample 292, harbouring only C1 according to the uncloned sample SSCP gel image, were sequenced and multiple variants of C1 and one C2 clone were detected. Only samples that contained clear, sharp SSCP gel image bands of type C2 or clade D or equal bands of both were chosen for direct sequencing (see below).

Comparison of phylotypes with other studies

Comparisons of the *Symbiodinium* clade C type nomenclature between studies is often hampered by the use of different parts of the nrDNA region. To overcome this, the entire *Symbiodinium* nrDNA-ITS region (comprising ITS1, 5.8S and ITS2) of a small subset of five uncloned type C1 and C2 (from ITS1 genotyping) samples from the tagged colonies was amplified (three sampled before bleaching and two sampled after bleaching) and sequenced as described by Baillie *et al.* (2000). Forward primer, ‘zITSf’: 5'- CCG GTG AAT TAT TCG GAC TGA CGC AGT GCT-3' (Hunt*er et a*l. 1997) and reverse primer ‘zITSr’: 5'- TCC TCC GCT TAT TGA TAT GC-3' (Whi*te et a*l. 1990) were used to obtain approximately 650 base pair PCR products (Table 1). Sequences were compared and aligned to previously sequenced ITS1 samples and closely matching sequences in Genbank using BioEdit (version 7.0) and CLUSTER W (version 1.7, Thomps*on et a*l. 1994).

The uncloned full-length ITS sequences were assigned to type C1 or C2 based on their match to the ITS1 region of the previously sequenced representative ITS1 region samples (Table 1) and the SSCP gel profile. The sequences were similar but not identical to the LaJeunesse ITS2 sequence regions of *Symbiodinium* type C1e & f (Genbank accession numbers AY258489, AY258490) found in *Fungia* *scutaria* (Hawaii LaJeunes*se et a*l. 2004), C1b (AY239363) and C1c (AY239364) from the Great Barrier Reef (2003) and to the entire ITS sequence region of *Symbiodinium* *goreaui* type C1 (AF333515 LaJeunesse 2001). The alignment of the full length ITS sequences from this study and the LaJeunesse C1 sequence (AF333515) consisted of six taxa and 542 alignment positions. Nine alignment positions were variable, 7 of which were parsimony informative. Phylogenetic analysis of the six full-length ITS sequences obtained from before and after the bleaching and the *Symbiodinium* *goreaui* C1 sequence (AF333515) confirmed the assignment to types C1 and C2 (Fig. 2c).

Twenty sequences have been submitted to Genbank under the accession numbers EU189435 - EU189455 (Table 1). All except one cloned and one uncloned ITS1 sequence had at least one or two base pair variations from previous Genbank entries. All sequences used as references, standards and matches from Genbank are shown in Table 2.

References

Baillie BK, Belda-Baillie CA, Maruyama T (2000) Conspecificity and Indo-Pacific distribution of *Symbiodinium* genotypes (*Dinophyceae*) from giant clams. Journal of Phycology 36: 1153-1161

Berkelmans R (2006) Bleaching and mortality thresholds: how much is too much? AIMS, Townsville

Berkelmans R, van Oppen MJH (2006) The role of zooxanthellae in the thermal tolerance of corals: a ‘nugget of hope’ for coral reefs in an era of climate change. Proceedings of the Royal Society of London Series B, Biological Sciences (1934-1990):

Fabricius KE, Mieog JC, Colin PL, Idip D, van Oppen MJ (2004) Identity and diversity of coral endosymbionts (zooxanthellae) from three Palauan reefs with contrasting bleaching, temperature and shading histories. Molecular Ecology 13: 2445-2458

Hunter CL, Morden CW, Smith CM (1997) The utility of ITS sequences in assessing relationships among zooxanthellae and corals. Proceedings of the 8th International Coral Reef Symposium, Panama 2: 1599-1602

LaJeunesse TC (2001) Investigating the biodiversity, ecology and phylogeny of endosymbiotic dinoflagellates in the genus *Symbiodinium* using the ITS region: in search of a "species" level marker. Journal of Phycology 37: 866-880

LaJeunesse TC, Loh WKW, van Woesik R, Hoegh-Guldberg O, Schmidt GW, Fitt WK (2003) Low symbiont diversity in southern Great Barrier Reef corals, relative to those of the Caribbean. Limnology and Oceanography 48: 2046-2054

LaJeunesse TC, Thornhill DJ, Cox EF, Stanton FG, Fitt WK, Schmidt GW (2004) High diversity and host specificity observed among symbiotic dinoflagellates in reef coral communities from Hawaii. Coral Reefs 23: 596-603

Saitou N, Nei M (1987) The neighbour-joining method: a new method for reconstructing phylogenetic trees. Molecular Biology and Evolution 4: 406-425

Thompson JD, Higgins DG, Gibson TJ (1994) ClustalW: Improving the sensitivity of progressive multiple sequence alignment through sequence weighting, position specific gap penalties and weight matrix choice. Nucleic Acids Research 22: 4673-4680

van Oppen MJH, Palstra FP, Piquet AMT, Miller DJ (2001) Patterns of coral-dinoflagellate associations in *Acropora*: significance of local availability and physiology of *Symbiodinium* strains and host-symbiont selectivity. Proceedings of the Royal Society of London Series B, Biological Sciences (1934-1990) 268: 1759-1767

White TJ, Bruns T, Lee S, Taylor WJ (1990) Amplification and direct sequencing of fungal ribosomal genes for phylogenetics. In: Innis MA, Gelfanfd DH, Sninsky JJ, J WT (eds) PCR Protocols: A Guide to Methods and Applications. Academic Press, San Diego, p 315-322

Table 1. Samples from which ITS1 and full ITS DNA sequences were obtained in this study via either direct sequencing of the PCR product (‘direct’) or cloning of the PCR product followed by sequencing of a number of clones (‘cloned’). Phylogenetic analysis of the ITS1 and full length ITS regions of a subset of the samples confirmed the presence of clade D and type C1 and C2 *Symbiodinium*. Sequenced samples were used as references in subsequent SSCP analysis to confirm the predominant symbiont type of the colonies in this study. Note, samples are classified by a prefix that represents the reef name (e.g. M= Miall Island, K=Keppels) and the colony tag number, and suffixed with a dash and a number if cloned.

| Inter-transcribed Spacer Region 1 (ITS1) | | | Full length Inter-transcribed Spacer Region  (ITS, comprising ITS1, 5.8S and ITS2) | |
| --- | --- | --- | --- | --- |
| Type C | | Type D | Type C | |
| Before bleaching | After bleaching | Before bleaching | Before bleaching | After bleaching |
| cloned | cloned | cloned | uncloned | uncloned |
| M20-3 EU189440 | M292-1 EU189447 | M283-1 EU189451 | M365 EU189438 | M365 EU189435 |
| M20-8 EU189441 | M292-2 EU189448 | M283-5 EU189452 | M392 EU189436 | M296 EU189439 |
| M20-11 EU189442 | M296-1 EU189449 | M283-11 EU189453 | M265 EU189437 |  |
|  | uncloned | M283-12 EU189454 |  |  |
|  | M291 EU189443 | M283-13 EU189455 |  |  |
|  | M296 EU189444 | uncloned |  |  |
|  | M349 EU189445 | M071 EU189450 |  |  |
|  | M2 EU189446 |  |  |  |

Table 2. Additional *Symbiodinium* types whose full ITS and ITS1 sequences were referred to in this study.

| Species | Clade | Host | Location | Citation | Accession No. |
| --- | --- | --- | --- | --- | --- |
| *Symbiodinium sp.* | C1 | *A. tenuis* | Magnetic Island | (van Opp*en et a*l. 2001) | AF380555 |
| *Symbiodinium sp.* | C2 | *A. millepora* | Orpheus Island | (van Opp*en et a*l. 2001) | AF380549 |
| *Symbiodinium sp.* | D | *A. millepora* | Magnetic Island | (van Opp*en et a*l. 2001) | EU024793 |
| *Symbiodinium sp.* | C | *A. millepora* | Keppel Islands | (Berkelmans 2006) | AY643496 |
| *Symbiodinium sp.* | D | Various spp. | Palau | (Fabrici*us et a*l. 2004) | AY457965 |
| *Symbiodinium*  *goreaui* | C1 | *Rhodactis* *lucida* | Carribean,  Jamaica | (LaJeunesse 2001) | AF333515 |
| *Symbiodinium sp* | C1b, c | Various spp. | Heron Island | (LaJeunes*se et a*l. 2003) | AY239363/4 |
| *Symbiodinium sp* | C1e | Various spp*.* | Hawaii | (LaJeunes*se et a*l. 2004) | AY258489 |
| *Symbiodinium sp.* | C1f | *Fungia scutaria* | Hawaii | (LaJeunes*se et a*l. 2004) | AY258490 |

Fig 1. Several SSCP variants of *Symbiodinium* types C and D were found before and after bleaching in *Acropora millepora* colonies from the Keppels. Cloning and sequencing of one of these (D*) revealed a difference of only one base pair from other type D sequences. Clade C variants that matched C1 and C2 had either none or 3 to 6 base pair differences.

Fig 2 a-c. Phylogenetic relationships derived by Maximum Parsimony analysis of **(a)** the clade C ITS1 and **(**b**)** clade D ITS1 and **(**c**)** clade C full length ITS regions of *Symbiodinium* sequences from *A. millepora* colonies in the Keppel region and matches from GenBank. (Trees are branch-length informative and numbers at nodes represent bootstrap support values based on 1000 replicates). Two major clades were identified using this method, C and D (van Opp*en et a*l. 2001). Standards with Genbank accession numbers used in SSCP analysis are shown in boldface. Branch lengths represent phylogenetic distances. Host names are shown in italics followed by the name of the reef. Type C samples taken before the bleaching are marked with ►. Subclades are labelled on the right. Cloned sequences are labelled with a dash following the sample number. M=Miall Island or Magnetic Island, K=Keppel islands.

a. Rooted *Symbiodinium* ITS1 type C (MP) phylogenetic relationships (1000 replicates) showing branch lengths to scale (Mega 3).

b. Rooted *Symbiodinium* ITS1 type D (NJ) phylogenetic relationships (1000 replicates). No type D samples were sequenced after the bleaching in January 2006. Branch lengths indicate the strength of the relationships. *denotes type D* which had a higher SSCP band compared to all other clade D sequences.

c. Rooted *Symbiodinium* full length nrDNA-ITS type C sequence (MP) consensus phylogenetic relationships (1000 replicates).

Figure 3. SSCP gel image showing diversity of symbiont types within a single sample after recovery from bleaching. The wide band of the uncloned sample 292 (far left) suggests multiple type C1 variants. Cloning and sequencing revealed 4 different sequences, unexpectedly including C2. The sample was taken after the colony bleached and then recovered. The SSCP image of the sample taken before bleaching showed a sharp C2 band with no obvious evidence of other background symbiont types. Greater than 90% of the colony (mostly the tips of branches) had mortality in August 2006, six months after bleaching.

Fig 1

Fig 2 a

Fig 2 b

Fig 2 c

C1

C2

Fig 3
